# Evaluating deep learning auto-contouring for lung radiation therapy: A review of accuracy, variability, efficiency and dose, in target volumes and organs at risk^☆^

**DOI:** 10.1016/j.phro.2025.100736

**Published:** 2025-02-21

**Authors:** Keeva Moran, Claire Poole, Sarah Barrett

**Affiliations:** Applied Radiation Therapy Trinity, Trinity St. James’s Cancer Institute, Discipline of Radiation Therapy, Trinity College, Dublin, Ireland

**Keywords:** Deep learning, Artificial Intelligence, Lung cancer, Delineation, Auto-contouring

## Abstract

**Background and purpose:**

Delineation of target volumes (TVs) and organs at risk (OARs) is a resource intensive process in lung radiation therapy and, despite the introduction of some auto-contouring, inter-observer variability remains a challenge. Deep learning algorithms may prove an efficient alternative and this review aims to map the evidence base on the use of deep learning algorithms for TV and OAR delineation in the radiation therapy planning process for lung cancer patients.

**Materials and methods:**

A literature search identified studies relating to deep learning. Manual contouring and deep learning auto-contours were evaluated against one another for accuracy, inter-observer variability, contouring time and dose-volume effects. A total of 40 studies were included for review.

**Results:**

Thirty nine out of 40 studies investigated the accuracy of deep learning auto-contours and determined that they were of a comparable accuracy to manual contours. Inter-observer variability outcomes were heterogeneous in the seven relevant studies identified. Twenty-four studies analysed the time saving associated with deep learning auto-contours and reported a significant time reduction in comparison to manual contours. The eight studies that conducted a dose-volume metric evaluation on deep learning auto-contours identified negligible effect on treatment plans.

**Conclusion:**

The accuracy and time-saving capacity of deep learning auto-contours in comparison to manual contours has been extensively studied. However, additional research is required in the areas of inter-observer variability and dose-volume metric evaluation to further substantiate its clinical use.

## Highlights

• Deep learning auto-contours have comparable accuracy to manual contours.
• Time saving compared to atlas auto contouring is reported for target volumes and organs at risk.
• Impact on inter-observer variability and dose was less frequently reported in studies.

## 1 Introduction

Accurate delineation of target volumes (TVs) and organs at risk (OARs) is a prerequisite for precise lung radiation therapy to produce optimal treatment plans [1]. TV delineation in lung cancer has higher delineation uncertainty than other cancer sites due to the complex tumour morphology and lack of contrast with adjacent normal tissues in the mediastinum [2]. Accurate OAR delineation helps to minimise normal tissue complication probability, reducing treatment toxicities and ensuring improved quality of life for patients.

TV and OAR delineation is a process that may take several hours due to the large anatomical area of the thorax and the resultant number of axial CT slices [3]. The observer’s expertise level greatly affects the quality of the contours [4] and inconsistencies can arise between observers during manual delineation resulting in inter-observer variability [5]. The latter is particularly relevant in the case of clinical target volume (CTV) delineation where clinician judgement is relied upon heavily, however, significant inter-clinician variations have been reported, even among experts [6]. Such differences in TV and OAR delineation negatively impact dose volume histogram (DVH) calculation and, consequently, tumour control probability and normal tissue complication probabilities [7]. Atlas-based auto segmentation algorithms have been widely adopted in lung radiation therapy to address the labour-intensive process of manual delineation, as well as improve inter-observer consistency [8], [9]. However, such algorithms are not fully automated and do not consistently provide accurate contours [10]. Neural network based deep learning algorithms, a form of data-driven artificial intelligence, have emerged in the literature as a promising solution to overcome the limitations of atlas-based mechanisms [11]. Deep learning has demonstrated significant potential in lung radiation therapy for TV and OAR delineation because the contours produced by such software have been shown to be more accurate with increased time saving compared to atlas-based contours [12]. Furthermore, as a result of automation, they require less manual input from experienced observers [12].

Deep learning segmentations are generated through the training, validation, and testing of deep learning algorithms using patients’ simulation scans. A number of deep learning algorithms have been tested in lung radiation therapy delineation, the most common being U-Net. This is a fully convolutional network (a CNN without fully connected layers) proposed by Ronneberger et al. [13] that is considered the gold standard for medical image delineation [3].

The primary benchmark for deep learning auto-contours is manual delineation. However, it is time consuming and subject to inter-observer variability, and therefore, alternative benchmarks are occasionally used. In relation to variability, Zhong et al. [14] and Zhu et al. [15] adopted the simultaneous truth and performance level estimation (STAPLE) algorithm in lung delineation. This is a weighted voting algorithm that uses the reference generated from multiple experts [16].

Deep learning algorithms have been developed in order to overcome the aforementioned limitations associated with manual delineation. In this context, the efficacy of deep learning algorithms in comparison to manual delineation requires analysis. This is achieved through assessing the accuracy, inter-observer variability, time, and dose-volume effects associated with deep learning auto-contours.

This review evaluated these outcome measures and assessed the performance of deep learning algorithms for their validation in clinical practice.

## 2 Material and methods

Review of the literature was conducted on “PubMed” and “Embase” using the search terms: “deep learning” OR “neural” AND “contour” OR “delineate” AND “lung” AND “tumor” OR “tumour” OR “organ at risk” OR “target”. Truncations were used on the search terms “contour”, “delineate”, “tumor”, “tumour”, “organ at risk” and “target” to maximise search output.

Randomised control trials, prospective, retrospective, and observational studies comparing and assessing deep learning auto-contouring to manual contouring were included in this review. Studies that examined the use of deep learning auto-contouring in lung cancer radiation therapy volume delineation were included. TVs of gross target volume (GTV) and CTV were eligible, and OARs of left lung, right lung, combined lung, heart, oesophagus, spinal cord, trachea, and mediastinum were included. Patients with different histological types, stages, and locations of lung cancer were included. No restrictions were placed on age, gender, or performance status. For studies reviewing OAR delineation, participants were permitted to have other thoracic cancers, provided part of the patient cohort had lung cancer. Excluded studies were those not reviewing the use of deep learning auto-contouring in the radiation therapy treatment planning process. In addition, studies that reported on non-artificial intelligence methods of auto-contouring were also excluded. Non-English language studies, or conference abstracts were excluded. Inclusion and exclusion criteria were designed to capture a broad range of clinically relevant papers on this topic.

Delineation on one or more imaging modalities comprising of computed tomography, magnetic resonance imaging, and positron emission tomography were included. No exclusion criteria were placed on the type of deep learning algorithms used.

**Fig. 1:**
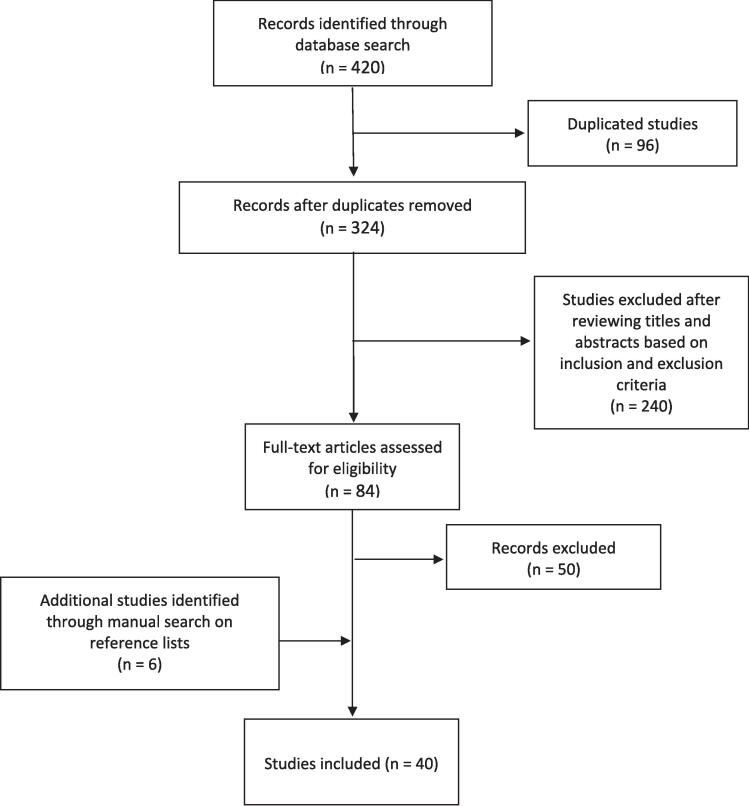
.

The final search was conducted on May 29th 2024, and the search strategy yielded 420 articles. Once duplicates were removed, 324 studies remained. After reviewing titles and abstracts based on the aforementioned inclusion and exclusion criteria, 84 studies were identified. Full texts of the studies were reviewed, and additional studies were detected through screening studies’ reference lists. Forty studies [2], [3], [7], [10], [11], [12], [14], [15], [17], [18], [19], [20], [21], [22], [23], [24], [25], [26], [27], [28], [29], [30], [31], [32], [33], [34], [35], [36], [37], [38], [39], [40], [41], [42], [43], [44], [45], [46], [47], [48] were included in this review with 13 reviewing TVs solely, 26 reviewing OARs solely, and one reviewing TVs and OARs. A summary of the PRISMA search strategy is detailed in Fig. 1. Of these 40 studies, seven articles do not have their data publicly available [2], [3], [18], [20], [40], [41], [46].

In total, approximately 9,000 patients (ranging from 9 to 2,208 per study) were used for training, validation, and end-testing of the deep learning algorithms across selected studies. Patients had stage I-IV lung cancers. The types of deep learning networks utilised were U-Net (n = 24) [2], [3], [10], [14], [15], [18], [19], [20], [21], [22], [26], [28], [29], [30], [35], [36], [37], [42], [43], [44], [45], [46], [47], [48], Res-Net (n = 6) [7], [17], [32], [37], [38], [47], commercial-based software (n = 6) [11], [12], [24], [25], [33], [39], DenseNet (n = 2) [29], [36], V-net (n = 2) [34], [40], A-net (n = 1) [25], and DPMS-R-CNN (n = 1) [21]. A summary of selected studies is provided in Supplementary Table 1.

The outcomes used to measure the effectiveness of deep learning auto-contouring methods were accuracy, inter-observer variability, contouring time, and dose-volume metric evaluation. Accuracy was quantified using the Dice Similarity Coefficient (DSC), a spatial overlap metric that quantifies the degree of overlap, and therefore, similarity between deep learning and manual contours. The DSC index was reported in all studies measuring accuracy and the variations used were the DSC and mean DSC. As DSC is sensitive to absolute volume, the Hausdorff distance, a distance metric that calculates the maximum Euclidean distance between the closest outer surface points of two contours [11], was also used to quantify accuracy. A Hausdorff distance of 0 mm indicates perfect surface contour correlation, while larger values represent increasing surface contour errors [11]. Hausdorff distance is sensitive to outliers, therefore, using the quantile method, the q^th^ quantile of distances, as opposed to the maximum, helps to mitigate this [49]. The 95th percentile Hausdorff distance was the most commonly reported Hausdorff distance metric included in the studies for review with 16 studies utilising it, while 11 studies used the standard Hausdorff distance and an additional 2 used the mean slice-wise hausdorff distance. The magnitude of corrections required, and the clinical acceptance rate were also reviewed in the accuracy analysis. Inter-observer variability was quantified using variations of the DSC, normalisation scores (the conversion of an original score to a standard score), the coefficient of variation (CV) (a volume metric), and standard distance deviation (SDD) (a spatial metric). The CV was defined as the standard deviation (SD) divided by the mean TV volume of all observers for each patient contoured with each delineation method. The SDD measures the dispersion of the centroid distribution of TVs manually contoured, which represents the SD of the distance of each point from the mean centre [17]. The contouring time encompassed both the inference time (the time taken for the deep learning algorithm to contour a patient’s scan) and the time taken for contour adjustment. Inference time was reported in seconds, while the total contouring time was reported in minutes and hours. The dose-volume metric evaluation was commonly assessed using Dmax (maximum dose) and Dmean (mean dose) of the relevant structure.

## 3 Results

### 3.1 Accuracy

The accuracy of the contours was reported in 39 of the 40 studies [2], [3], [7], [10], [11], [12], [14], [15], [17], [18], [19], [20], [22], [23], [24], [25], [26], [27], [28], [29], [30], [31], [32], [33], [34], [35], [36], [37], [38], [39], [40], [41], [42], [43], [44], [45], [46], [47], [48]. Thirteen TV studies assessed the accuracy of deep learning auto-contours, while 26 OAR studies assessed this. A comprehensive review of studies is presented in Supplementary Table 2. The benchmarks for deep learning auto-contours used between studies were manual contours completed by one or more observers (n = 23) [17], [18], [22], [23], [24], [25], [26], [27], [29], [31], [33], [34], [35], [36], [37], [38], [48], manual contours delineated in accordance with guidelines (n = 14) [2], [3], [7], [10], [11], [12], [19], [20], [28], [30], [32], [41], [45], [47] and the STAPLE algorithm (n = 2) [14], [15]. Of the 14 studies that used guidelines, they were derived from the Radiation Therapy Oncology Group (RTOG) (n = 9) [3], [7], [10], [19], [20], [32], [41], [45], [47], institutional (n = 4) [2], [11], [12], [28], and the National Comprehensive Cancer Network (n = 1) [30].

Studies reviewing TVs reported DSC scores that ranged from 0.71 to 0.88 for GTVs and scores of 0.75 to 0.86 for CTVs (Table 1). Studies reviewing OARs reported DSC scores ranging from 0.94 to 1.0 for the left lung, 0.92 to 1.0 for the right lung, 0.95 to 0.99 for the combined lung, 0.85 to 0.96 for the heart, 0.55 to 0.87 for the oesophagus, 0.74 to 1.0 for the spinal cord, 0.81 to 0.93 for the trachea and 0.93 to 0.95 for the mediastinum (Table 2).

**Table 1:** Summary of Dice Similarity Coefficent and Hausdorff Distance Target Volume metrics reported.

**Study Author (Year), [Reference]**	**Target volume**	**Dice Similarity Coefficient**	**Hausdorff Distance**
Bi, N., et al. (2019) [17]	CTV	DSC: 0.75 ± 0.06	N/R
Hosny, A., et al. (2022) [21]	GTV	N/R	N/R
Lei, Y., et al. (2022) [23]	GTV	DSC: 0.84–0.88 ± 0.15	HD95: 3.06–4.6 mm
Wang, C., et al. (2019) [27]	GTV	DSC: 0.82 ± 0.10	N/R
Wang, S., et al. (2022) [2]	GTV	Mean DSC: 0.83 ± 0.07	HD: 5.9 ± 2.5 mm
Xie, Y., et al. (2021) [29]	CTV	DSC: 0.86 ± 0.03	HD: 5.5–8.6 mm
Yu X., et al. (2022) [30]	GTV	DSC: 0.74	HD95: 21.39 mm
Zhong Z., et al. (2019) [14]	GTV	DSC: 0.86 ± 0.04	N/R
Yu, X., et al. (2023) [36]	GTV	DSC: 0.84 ± 0.06	N/R
Zhang, F., et al. (2024) [37]	GTV	DSC: 0.80 ± 0.13	HD95: 9.43 mm
Primakov, S. P., et al.(2022) [43]	GTV	DSC: 0.85 ± 0.15	HD95: 5 mm
Shen, J., et al. (2022) [45]	CTV	DSC: 0.81	HD95: 1.61–4.68 mm
Kulkarni, C., et al.(2024) [42]	GTV	DSC: 0.77 (0.0–0.93)	N/R
Wong J., et al. (2021) [28]	GTV	Mean DSC: 0.71 (0.19–0.90)	HD95: 5.23 mm (2.04–15.17)

CTV: clinical target volume, GTV: gross target volume, DSC: dice similarity coefficient, HD: Hausdorff distance, HD95: 95th percentile Hausdorff distance,

**Table 2:** Summary of Dice Similarity Coefficent and Hausdorff Distance organ at risk metrics reported.

	**Dice Similarity Coefficient**								**Hausdorff Distance (mm)**							
**Study Author (Year), [Reference]**	LL	RL	CL	H	O	SC	Tr	M	LL	RL	CL	H	O	SC	Tr	M
Wong J., et al. (2021) [28]	0.98	0.98	N/R	0.95	0.81	0.90	0.91	N/R	2.93	3.04	N/R	5.09	3.32	1.62	2.27	N/R
Cao, Z., et al. (2021) [18]	0.97	0.97	N/R	0.95	0.85	0.91	0.81	N/R	N/R	N/R	N/R	N/R	N/R	N/R	N/R	N/R
Dong, X., et al. (2019) [19]	0.97	0.97	N/R	0.87	0.75	0.90	N/R	N/R	2.07	2.50	N/R	4.58	4.52	1.19	N/R	N/R
Feng, X., et al. (2019) [20]	0.98	0.97	N/R	0.93	0.73	0.89	N/R	N/R	2.10	3.96	N/R	6.57	8.71	1.89	N/R	N/R
Francis, S., et al. (2022) [3]	0.98	0.97	N/R	0.94	0.74	0.90	N/R	N/R	1.47	2.70	N/R	3.63	1.85	6.65	N/R	N/R
Johnston, N., et al. (2022) [22]	N/R	N/R	0.98	0.91	0.72	0.80	0.84	N/R	N/R	N/R	6.90	17.6	11.6	29.6	11.8	N/R
Lustberg, T., et al. (2018) [12]	0.98	0.98	N/R	0.93	0.76	0.83	N/R	0.95	3.0	4.0	N/R	14.0	6.0	4.0	N/R	7.0
Nemoto, T., et al. (2020) [10]	N/R	N/R	0.99	N/R	N/R	N/R	N/R	N/R	N/R	N/R	N/R	N/R	N/R	N/R	N/R	N/R
Vaassen, F., et al. (2022) [24]	1.0	1.0	N/R	0.89	0.73	1.0	N/R	0.93	0.9	1.1	N/R	11.3	3.2	0	N/R	9.9
Vaassen, F., et al. (2021) [25]	N/R	N/R	N/R	N/R	N/R	N/R	N/R	N/R	N/R	N/R	N/R	N/R	N/R	N/R	N/R	N/R
Vu, C. C., et al. (2020) [26]	0.97	0.97	N/R	0.90	0.64	0.75	N/R	N/R	4.0	5.1	N/R	9.8	9.2	9.5	N/R	N/R
Zhang, F., et al. (2022) [31]	0.95	0.96	N/R	0.86	0.67	0.89	0.91	N/R	6.47	6.09	N/R	9.75	6.14	2.05	2.44	N/R
Zhang, T., et al. (2020) [22]	0.95	0.94	N/R	0.89	0.73	0.82	N/R	N/R	N/R	N/R	N/R	N/R	N/R	N/R	N/R	N/R
Zhu, J., et al. (2019) [7]	0.95	0.96	N/R	0.91	0.75	0.87	N/R	N/R	N/R	N/R	N/R	N/R	N/R	N/R	N/R	N/R
Zhu, J., et al. (2019) [15]	N/R	N/R	0.95	0.91	0.64	0.76	N/R	N/R	N/R	N/R	7.96	7.98	9.25	8.74	N/R	N/R
Maduro Bustos, L. A., et al. (2023) [33]	0.95	0.96	N/R	0.95	0.75	0.83	N/R	N/R	12.5	12.0	N/R	10.1	21.0	4.20	N/R	N/R
Pan, S., et al. (2023) [34]	0.97	0.98	N/R	0.90	N/R	0.89	N/R	N/R	2.64	7.03	N/R	8.21	N/R	8.77	N/R	N/R
Saha, M., et al. (2023) [35]	0.96	0.97	N/R	0.94	0.61	N/R	N/R	N/R	0.8	0.7	N/R	2.0	1.0	N/R	N/R	N/R
Zhang, F., et al. (2023) [38]	0.94	0.92	N/R	0.89	0.73	0.87	0.81	N/R	10.77	11.92	N/R	8.89	0.73	0.87	0.81	N/R
Harten, L., et al. (2019) [41]	N/R	N/R	N/R	0.84	0.94	N/R	0.91	N/R	N/R	N/R	N/R	3.40	2.0	N/R	2.10	N/R
Vesal, S., et al. (2019) [46]	N/R	N/R	N/R	0.94	0.86	N/R	0.93	N/R	N/R	N/R	N/R	0.33	0.23	N/R	0.19	N/R
Han, M., et al. (2019) [40]	N/R	N/R	N/R	0.95	0.87	N/R	0.93	N/R	N/R	N/R	N/R	1.30	2.60	N/R	1.50	N/R
Gibbons, E., et al.(2023) [11]	0.98	0.98	N/R	0.96	0.74	N/R	N/R	N/R	18.9	17.0	N/R	17.6	12.4	N/R	N/R	N/R
Chen, W., et al.(2021) [39]	0.98	0.98	N/R	0.93	0.75	0.90	N/R	N/R	N/R	N/R	N/R	N/R	N/R	N/R	N/R	N/R
Ribeiro, M., et al.(2023) [44]	0.96	0.96	N/R	0.94	0.78	N/R	N/R	N/R	1.40	1.60	N/R	1.80	1.20	N/R	N/R	N/R
Yang, J., et al.(2018) [47]	0.95–0.98	0.95–0.97	N/R	0.85–0.93	0.55–0.72	0.83–0.89	N/R	N/R	2.9–7.8	4.1–14.5	N/R	5.8–13.8	7.3–37.0	1.9–8.1	N/R	N/R
Mehta, A., et al.(2023) [48]	0.94	0.92	N/R	0.88	N/R	0.74	N/R	N/R	3.6	4.1	N/R	4.2	N/R	2.85	N/R	N/R

mm: milimetres, N/R: not reported, LL: left lung, RL: right lung, H: heart, O: oesophagus, SC: spinal cord, Tr: trachea, M: mediastinum, CL: combined lung.

The calculated mean Hausdorff distances for GTVs and CTVs were 7.8 mm and 6.6 mm respectively (Table 1). OARs achieved mean Hausdorff distances of 5.2 mm for the left lung, 5.9 mm for the right lung, 7.2 mm for the combined lung, 7.7 mm for the heart, 8.1 mm for the oesophagus, 5.9 mm for the spinal cord, 3.4 mm for the trachea and 8.5 mm for the mediastinum. An overview of the quantitative evaluation metric results can be found in Table 2. Studies that obtained the highest DSC scores for the GTV, CTV, left lung, right lung, oesophagus, spinal cord, and trachea, used standard manual contours as the benchmark [23], [24], [29], [40]. Using standard manual contours as a benchmark may have resulted in a greater accuracy of deep learning auto-contours because deep learning algorithms are predominantly trained on cases that are manually delineated in the absence of guidelines or randomisation algorithms such as the STAPLE [29].

The highest DSC score and lowest HD achieved for TVs was acquired using the hybrid DPMS-R-CNN algorithm which may be due to its possible improved delineation capacity from the five subnetworks cascaded in (as opposed to the typical one subnetwork) [23], or a reflection of the disease stage investigated in this paper which was early stage NSCLC. A commercial deep learning algorithm performed best for OAR delineation as it achieved the highest DSC scores for the left lung, right lung, heart, spinal cord, and mediastinum [11], [25], [26]. This is likely due to its good performance considering its use in clinical practice.

Seven studies assessed the accuracy of deep learning auto-contours by determining whether they were suitable for clinical use via observers’ subjective judgements. In Wang et al. [2] radiation oncologists categorised each GTV deep learning auto-contour and manual contour as accepted, accepted with modifications, or rejected for 20 patient cases. They found that 88.8 % versus 91.3 % of segmentations for deep learning and manual contours respectively, were accepted or accepted with modification. The authors noted that the modifications were relatively minor for both the deep learning and manual contours. Hosny et al. [23] conducted a similar analysis for GTVs. Out of 80 cases, 79 % of the deep learning auto-contours were rated as “acceptable with minor modifications”. Shen et al [45] conducted a similar evaluation and 98.9 % versus 98.1 % of deep learning and manual contours respectively were deemed acceptable. Primakov et al. [43] stated that participants preferred the deep learning auto-contours to expert’s manual contours in 55 % of cases. In Maduro et al. [33] a radiation oncologist rated deep learning auto-contours for OARs in 10 patient cases and categorised them as clinically acceptable, minor edits, major edits and must redo. They found that 96 % of delineations were deemed clinically usable or required minor edits, while 4 % required major edits or had to be redone, which was for the oesophagus. Gibbons et al. [11] obtained similar results whereby 69.2 % of deep learning auto-contours were observed as clinically acceptable, while the oesophagus required major edits. In Ribeiro et al. [44], a radiation oncologist graded 85 % of deep learning auto-contours as at least ‘ready to use’, while only 65 % of manual contours received this grade.

Eight studies conducted additional evaluation of the accuracy of deep learning auto-contours using atlas-based contours for comparison [10], [11], [12], [15], [26], [32], [39], [47]. All studies found that the deep learning auto-contours outperformed atlas-based contours. Deep learning algorithms have a greater ability to overcome variations in patient anatomy as a result of learning complex non-linear relationships within imaging data, [12], thus resulting in superior auto-contours.

Overall, deep learning algorithms yielded higher DSC values for the majority of OAR segmentations in comparison to TVs [3], [17], [19], [24], [28], [30], [34].

### 3.2 Inter-observer variability

Seven studies reported on the inter-observer variability of delineation methods [7], [15], [17], [20], [21], [43], [47]. Of these, three TV studies assessed whether deep learning algorithms were associated with reduced inter-observer variability, while four OAR studies assessed this. The studies are detailed in Supplementary Table 2. The metrics used to assess inter-observer variability were DSC (n = 4) [7], [15], [21], [43], normalisation scores (n = 2) [20], [47], surface DSC (n = 1) [21], CV (n = 1) [17] and SDD (n = 1) [17].

Two of the three TV studies found a notable reduction in inter-observer variability when deep learning algorithms were used [17], [21]. Bi et al. [17] measured the inter-observer variability between 11 radiation oncologists’ manual contours and deep learning auto-contours using the CV and SDD. A larger CV and SDD indicated greater variability or lower consistency. A 35 % reduction in SDD and a 30 % decrease in CV were achieved with deep learning auto-contours in comparison to manual contours, thus reducing inter-observer variability. The TV in this study was a post-operative CTV, which may inherently be more challenging than a definitive case, therefore leading to the increased inter-observer variability among experts. Hosny et al. [21] used the DSC and surface DSC to measure inter-observer variability between the manual contours and deep learning auto-contours of 6 observers. A DSC of 0.91 and surface DSC of 0.86 were achieved using deep learning algorithms in comparison to the manual inter-observer benchmark of 0.83 and 0.72, yielding a stated 32 % reduction in inter-observer variability. Primakov et al. [43] conducted a similar analysis using the DSC to assess inter-observer variability between seven medical imaging specialists for 25 patients. However, the proposed method achieved an average DSC of 0.82, while the average DSC of experts’ inter-observer variability was 0.84, indicating a slight increase in variability for the deep learning auto-contours.

Four OAR studies assessed inter-observer variability and obtained differing results [7], [15], [20], [47]. Feng et al. [20] reviewed three experts’ contours in relation to three patients. Normalisation scores were generated for each OAR and averaged over the three cases. Inter-observer variability was given a reference score of 50 based on manual contours. The deep learning algorithm achieved scores of 80, 71, 61, 47, and 28 for the left lung, right lung, spinal cord, heart, and oesophagus respectively, thus showing reduced inter-observer variability for the lungs and spinal cord. The heart yielded similar results to manual delineation, and the oesophagus performed significantly worse.

Yang et al. [47] also assigned each OAR with a normalisation score for five deep learning methods and two atlas-based methods. Deep learning methods one to three achieved overall normalisation scores above 50, indicating reduced inter-observer variability, however, deep learning methods five and seven and both atlas-based methods received overall scores below 50, showing increased variability. All methods achieved scores below 50 for the heart and oesophagus.

Zhu et al. [7] analysed the inter-observer variability based on three experts’ delineations in relation to twelve patients. Unlike Feng et al., this was achieved through examining significant differences between experts and the deep learning algorithms using 95 % confidence intervals of DSCs. Significant differences were obtained for the heart and oesophagus when the deep learning algorithm was used, indicating increased inter-observer variability. However, no significant differences were associated with the lungs and spinal cord, indicating no improvement in variability. The results of this study were similar to a previous study conducted by Zhu et al. [15] which utilised the aforementioned methodology. There was no improvement in inter-observer variability for the lungs, spinal cord, and heart, while the oesophagus was associated with increased variability when a deep learning algorithm was used.

### 3.3 Contouring time

The contouring time was reported in 24 of 40 studies [3], [7], [11], [12], [15], [17], [18], [19], [20], [21], [26], [27], [29], [30], [31], [32], [33], [34], [35], [36], [40], [43], [45], [47], eight of which were TV studies and sixteen of which were OAR studies. A summary of details is provided in Supplementary Table 2. The methodologies between studies used to measure contouring time were consistent with one another, thus facilitating ease of comparison. The inference time was reported in eighteen of the studies [3], [15], [18], [19], [20], [26], [27], [29], [30], [31], [32], [34], [35], [36], [40], [43], [45], [47]. Fourteen studies [3], [15], [18], [19], [26], [29], [30], [32], [34], [35], [36], [40], [43], [47] reported inference times without reporting the times required to manually adjust the deep learning auto-contours. Inference times ranged from 3.8 s to 1.6 min, with one outlier totalling a median inference time of nine minutes [35]. This outlier was due to the heart substructures also being segmented which take significantly longer to contour due to their complexity. The absence of information regarding the time required to manually adjust the deep learning auto-contours in the 14 studies that solely reported the inference time makes it difficult to determine the overall contouring time. This is because it is the entire delineation process, from contour generation to user adjustment that is indicative of the actual time saving associated with deep learning algorithms.

Three studies also reported the inference times of atlas-based algorithms [15], [32], [47]. Atlas-based algorithms produced inference times of 2.4 to 5 min, with an outlier of 8 h. The inference time for deep learning algorithms is generally significantly faster than for atlas-based because the process of transforming CT images into atlas images is highly time consuming [50]. A trained CNN, however, can quickly accomplish automatic segmentation without requiring any changes to the CT images [32].

Nine studies [7], [11], [12], [17], [20], [21], [27], [31], [45] reported the total time taken to produce and edit the deep learning auto-contours. The total contouring time required ranged from 5.4 min to 30 min for TVs, while OARs had a time range of 6.7 min to 20 min. Eleven studies [7], [11], [12], [17], [20], [21], [29], [31], [32], [43], [45] recorded the time taken to produce manual contours, resulting in time ranges of 2.9 to 80 min for TVs and 10.3 to 50 min for OARs. One additional study [33] reported the total time saved, without reporting the time taken for manual or deep learning auto-contouring. The aforementioned studies found that deep learning algorithms were associated with a significant time saving in comparison to manual delineation with reductions between 35 % to 84 %. Lustberg et al. [12] and Gibbons et al. [11] also evaluated the time taken to produce and adjust deep learning auto-contours in comparison to atlas-based contours for OARs. These studies demonstrated that deep learning auto-contours were associated with an 18 %-19 % time saving in comparison to atlas-based contours. Hosny et al. [21] conducted a qualitative analysis where observers were surveyed on whether they thought the deep learning auto-contours improved efficiency due to the time reduction. The observers agreed that 77 of 80 deep learning auto-contours led to increased efficiency.

### 3.4 Dose volume metric evaluation

Eight of 40 studies [3], [19], [21], [22], [25], [31], [35], [38] reported the impact of deep learning auto-contours on radiation therapy treatment plans using dose-volume metrics. Seven of these studies were OAR studies, while only one was a TV study. Metrics used to quantify the dose-volume differences were the mean dose (Dmean) (n = 7) [3], [19], [22], [25], [31], [35], [38], maximum dose (Dmax) (n = 6) [3], [19], [25], [31], [35], [38], Vx (n = 6) [3], [21], [22], [25], [31], [38], Dose to 95 % (D95) (n = 2) [19], [21], Dose to 50 % (D50) (n = 2) [19], [35], Dose to 5 % (D5) (n = 2) [19], [35], minimum dose (Dmin) (n = 1) [19], Dose to 2 % (D2) (n = 1) [22], and V95 (n = 1) [21]. D95/50/5/2 is the dose being received by 95/50/5/2% of the TV or OAR. Vx is the percentage of the OAR volume receiving ≥ xGy. V95 is the percentage of the TV receiving at least 95 % of the prescription dose.

All eight studies used differing methodologies to present and analyse the data. However, six of the studies used p-values to determine whether there were statistically significant dose differences between manual contours and deep learning auto-contours [19], [21], [22], [31], [35], [38]. A p-value of greater than 0.05 indicated no statistically significant difference. All six studies reported no clinically significant differences in the dose-volume effect of contour variations between manual contours and deep learning auto-contours for TVs and OARs on patients’ treatment plans. Three studies reported p-values all greater than 0.05, indicating a lack of statistically significant dose-volume differences [21], [31], [38]. The remaining three studies that used p-values reported some statistically significant differences; however, none were deemed to be clinically significant. Of these, Dong et al. [19] reported 6 of 20 plans had statistically significant dose differences with p-values less than 0.05, however, the actual dose differences for OARs were 0.03 to 0.2 Gy. Such differences are negligible because the clinically acceptable dose and volume differences between manual and automatic contours are below 1 Gy and 1 %, respectively [31]. Johnston et al. [22] obtained statistically significant differences for the Lung Dmean and V20, however, the actual dose differences were very low at less than 0.3 Gy and 0.61 %, therefore, the results were not clinically significant. Saha et al. [35] also reported statistically significant differences between manual and deep learning auto-contours for the heart, lungs and oesophagus. However, the median absolute difference in mean dose to these OARs was < 0.1 Gy, indicating clinical insignificance.

The remaining two studies that did not use p-values to establish statistically significance dose differences between manual contours and deep learning auto-contours also determined that there was no clinical significance in such differences. Vaassen et al. [25] reported dose differences greater than 1 Gy for the heart Dmean when there was overlap between this OAR and the planning target volume. These differences were also deemed to be clinically insignificant by the study’s authors. Francis et al. [3] demonstrated that although the contour volumes between manual contours and deep learning auto-contours differed slightly, the impact on the dose distribution was negligible.

Only one study involved in dose-volume evaluation was prospective in nature [25]. As such, the true clinical impact of dose-volume differences resulting from contour variations between manual contours and deep learning auto-contours could not be analysed in practice. The results presented above should, therefore, be treated with a degree of caution.

## 4 Discussion

Manual delineation of TVs and OARs in lung radiation therapy is subject to inaccuracy and variability, and the process is highly tedious and labour intensive [3]. The use of deep learning algorithms has been investigated in the forty studies included in this review for their appropriateness as an alternative to manual delineation and to assess the potential benefits associated with their use, the primary one being time saving.

Deep learning algorithms have been shown to have a comparable accuracy to that of manual delineation for TVs and OARs, with thirty-four of thirty-nine studies reporting DSC scores above 0.7. The remaining five studies [15], [26], [31], [35], [47] yielded DSC scores below 0.7 for the oesophagus solely, highlighting that improvements are required in this challenging structure. DSC alone is a limited indication of contour suitability due to the impact of absolute volume on this metric, therefore it should be considered in conjunction with additional metrics such as HD. This poor performance for the oesophagus was also reflected in the HD values as this OAR obtained the highest HD of 37 mm [47] and the second highest overall HD average of 8.06 mm. This may be due to poor visualisation of the oesophagus because of its low soft tissue contrast, irregular and large shape variability across patients, and indistinct boundary with surrounding soft tissues [15], [31]. As such, the oesophagus may be better suited for auto-contouring of every second or third slice and then edited manually, thus still contributing to clinical workflow [28]. An alternative solution may involve selecting different deep learning networks based on variable characteristics of OARs. Zhang et al. [36] demonstrated this as a different deep learning network was used for the oesophagus from the other OARs in order to increase its segmentation accuracy. Overall, deep learning algorithms yielded higher DSC values and lower HD values for the majority of OAR segmentations in comparison to TVs [3], [17], [19], [24], [28], [30], [34]. This may be due to the fact that the boundaries of OARs are typically more easily visualised than those of TVs. In addition, there is less anatomical variation in normal tissue i.e. OARs in comparison to tumours where such variation is significant. Tumour anatomical variation is particularly apparent in smaller tumours due to the irregular edges, large morphological differences, and random positions in comparison to large tumours which typically have smooth boundaries [37]. Consequently, there is a clear trend toward improved performance and less variability associated with deep learning algorithms for larger and less complex tumours [37], [43]. In relation to TV delineation, a confounding factor beyond the scope of this review is stage of disease. The study reporting the highest DSC and lowest HD for target volumes was in patients treated for early-stage NSCLC using a stereotactic approach [23]. These tumours tend to be small well circumscribed lesions, and prior data has demonstrated that auto-contouring performs better in lesions not abutting normal tissue [51].

Seven studies [7], [15], [17], [20], [21], [43], [47] assessed the inter-observer variability associated with deep learning algorithms in comparison to manual delineation. Of these, two studies determined that such algorithms are associated with a reduction in inter-observer variability when delineating TVs, while only one of the remaining studies reported a significant difference for OAR delineation. However, similar to accuracy outcomes, the oesophagus performed poorly in terms of inter-observer variability due to the same issues with indistinguishable boundaries and shape variability. The heart also performed poorly as the boundaries of this structure can be challenging to distinguish from surrounding normal tissue. Notwithstanding that the lungs and spinal cord are easily visualised structures; two studies noted no improvement in inter-observer variability for these OARs. This may be due to variability present in the training data which can lead to inaccuracies in the contours produced by software. Therefore, consensus should be reached between observers prior to training deep learning algorithms [12].

The 24 studies [3], [7], [11], [12], [15], [17], [18], [19], [20], [21], [26], [27], [29], [30], [31], [32], [33], [34], [35], [36], [40], [43], [45], [47] that measured the time saving associated with deep learning auto-contours in comparison to manual and/or atlas-based contours all demonstrated significant time reductions for deep learning auto-contours. The time saved using deep learning algorithms can be used, inter alia, to increase patient throughput in radiotherapy departments leading to improved patient care. Reduction in time consumption can also reduce workload for staff, thus creating a better work environment due to reduced levels of stress which are prominent in busy radiation therapy departments [52].

Only eight studies [3], [19], [21], [22], [25], [31], [35], [38] investigated the dose-volume metric evaluation of deep learning auto-contours on radiation therapy treatment plans. All studies reported that there were no clinically significant differences in comparison to manual contours. These findings are reassuring; however, clinical evaluation of auto-contouring is a valuable assessment to augment the geometric metrics of agreement and should be considered a key aspect of future auto-contouring studies. There is a lack of clarity and consensus on the plan optimisation strategy used in the studies that evaluated the dosimetric impact of AI generated contours. Some studies recalculated DVH metrics on the new contours and reported differences between the manual and auto-contours. Other studies reported plan optimisation on the new contours, and some did not report the detail on the methods employed. Future studies should more explicitly state their approach so meaningful comparisons can be made.

While numerous commercial deep learning algorithms exist for OARs, the same cannot be said for TVs. Many research deep learning algorithms are available for TV delineation, the most common being variations of the U-Net architecture, however, their use in clinic is limited for a number of reasons. Firstly, a deep learning algorithm trained in one radiation therapy clinic using data by their radiation oncologists (ROs) may not be transferable to another clinic among different ROs due to patient cohort differences and delineation variability between experts [42]. However, this limitation is not prominent for OARs as deep learning algorithms have been shown to perform well in this context [11]. Another major obstacle for deep learning TV segmentation acceptability is that a reliable ground truth does not exist because there is no unequivocal segregation of the TV from surrounding lung tissue. As such, every training example generated by ROs for deep learning is subjective to some extent [42]. Finally, deep learning algorithms for TV delineation do not perform as well as those for OAR segmentation, as shown by the DSC and HD values summarised in this review. TV studies recognise the limitations of their proposed algorithms and state that improvements are required prior to implementation in clinic [28], [30].

It is important to note the limitations of this review. Only two of 40 studies conducted prospective analyses. However, no studies included in this review analysed the clinical impact on patients (i.e. tumour control and normal tissue complication probabilities) from utilising deep learning algorithms for delineation in practice. This highlights the need for increased prospective validation of deep learning algorithms in clinical settings in order to evaluate treatment outcomes. An additional limitation is that while accuracy measures were extensively reported, fewer studies assessed inter-observer variability (seven studies) and dose-volume metric evaluation (eight studies). In order to minimise this impact, all literature that met the inclusion criteria were inserted into a data table to ensure a robust synthesis and mapping of the evidence base. In spite of such limitations, this study highlights the potential of deep learning algorithms in lung radiation therapy. While other reviews on this topic exist, they focus on the architecture of deep learning networks and lack a focus on the clinically relevant metrics of dosimetric reliability or time saving [53], [54].

In conclusion, evaluating the accuracy, inter-observer variability, time, and dose-volume metric impact is fundamental to determining the potential of deep learning algorithms for TV and OAR delineation in lung radiation therapy. Notwithstanding that broadly similar outcomes between deep learning auto-contours and manual contours were identified for accuracy, inter-observer variability, and dose-volume metric evaluation, significant time savings were identified in this review. Time saving is a key clinical metric often overlooked in the literature and while valuable in general, this could be particularly beneficial in the context of new developments such as adaptive radiotherapy, where producing contours in a timely manner is a necessity for its implementation.

## CRediT authorship contribution statement

**Keeva Moran:** Data curation, Methodology, Writing – original draft, Writing – review & editing. **Claire Poole:** Methodology, Supervision, Writing – review & editing. **Sarah Barrett:** Conceptualization, Methodology, Supervision, Writing – review & editing.

## Declaration of competing interest

The authors declare that they have no known competing financial interests or personal relationships that could have appeared to influence the work reported in this paper.
