# Supplementary material for: Evaluating deep learning auto-contouring for lung radiation therapy: A review of accuracy, variability, efficiency and dose, in target volumes and organs at risk
Source: Phys Imaging Radiat Oncol. 2025 Feb 21;33:100736. doi: 10.1016/j.phro.2025.100736 (PMC11914827; doi:10.1016/j.phro.2025.100736)
Supplement: Supplementary Data 1 [file mmc1.docx]

**Table 1: Summary of included studies**

| **Study Author (Year), [Reference]** | **Study type** | **Target volume or organ at risk** | **Deep learning algorithm** | **Number of Patients**  **(Training, validation, End-Testing)** | **Observers** | **Benchmark** |
| --- | --- | --- | --- | --- | --- | --- |
| Bi, N., et al. (2019) [17] | R | CTV | ResNet-101 | n= 269 NSCLC  (T= 200, V= 50, ET= 19) | 14 ROs | MC |
| Hosny, A., et al. (2022) [21] | R | GTV | 3D U-Net model | 2236 NSCLC  (T= 787, V= 1421, ET= 28) | 16:  14 ROs and  2 thoracic radiologists | MC |
| Lei, Y., et al. (2022) [23] | R | GTV | DPMS-R-CNN | 100 ES-NSCLC or lung metastases  (T= NR, V= NR, ET= NR) | 2 Physicians | MC |
| Wang, C., et al. (2019) [27] | P | GTV | 2D A-Net | 9 LA-NSCLC with involved mediastinal lymph nodes  (T= NR, V= NR, ET= NR) | 1 RO | MC |
| Wang, S., et al. (2022) [2] | R | GTV | 3D dual-modality based on U-Net and V-Net | 290 NSCLC  (T= 116, V= 58, ET= 58) | 6 ROs | MC using guidelines |
| Xie, Y., et al. (2021) [29] | R | CTV | 3D U-Net | 84 LA-NSCLC  (T= 40, V= 28, ET= 16) | Multiple | MC |
| Yu X., et al. (2022) [30] | R | GTV | 3D ResSE-U-Net | 214 NSCLC  (T= 148, V= 30, ET= 36) | Multiple | MC using guidelines |
| Zhong Z., et al. (2019) [14] | R | GTV | 3D U-Net | 60 NSCLC  (T= 38, V= 10, ET= 22) | 3 ROs | MC consensus using STAPLE algorithm |
| Yu, X., et al. (2023) [36] | R | GTV | Dual modality 3D U-Net based network | 86  (T= 54, V= 18, ET= 14) | Experienced Clinician(s) | MC |
| Zhang, F., et al. (2024) [37] | R | GTV | A two stage coarse and fine segmentation CNN network using U-Net and ResU-Net | 300  (T= 200, V= 40, ET= 60) | Multiple radiologists | MC |
| Primakov, S. P., et al.  (2022) [43] | R & P | GTV | 2D U-net | 1328  (T= 999, V= 236, ET= 93) | 40 Medical professionals | MC |
| Shen, J., et al. (2022) [45] | P | CTV | DiUNet | 200 stage III-IV SCLC  (T/V= 180, ET=20) | 2 ROs | MC using guidelines |
| Kulkarni, C., et al.  (2024) [42] | R | GTV | Canonical U-Net and Attention U-Net | 514 NSCLC  (T= 514, V= NR, ET=125) | 7 ROs | MC |
| Wong J., et al. (2021) [28] | R | GTV  LL, RL, H, O, SC, Tr | 3D U-Net | 310  (T= 210, V= 50, ET= 50) | Multiple | MC using guidelines |
| Cao, Z., et al. (2021) [18] | R | LL, RL, H, O, SC, Tr | 3D SE-ResU-net | 60  (T= 40, V= 10, ET= 10) | Multiple | MC |
| Dong, X., et al. (2019) [19] | R | LL, RL, H, O, SC | 3D U-Net-GAN | 55 SBRT  (T= 35, V= NR, ET= 20) | NR | MC using guidelines |
| Feng, X., et al. (2019) [20] | R | LL, RL, H, O, SC | 3D U-Net | 60  (T= 36, V= NR, ET= 24) | Multiple | MC using guidelines |
| Francis, S., et al. (2022) [3] | R | LL, RL, H, O, SC | ThoraxNet (3D U-Net-based) | 60  (T= 36, V= NR, ET= 24) | NR | MC using guidelines |
| Johnston, N., et al. (2022) [22] | R | CL, RL, H, O, SC, Tr | 3D U-Net | 105 LA-NSCLC or ES-NSCLC  (T= 55, V= NR, ET= NR) | 3: 2 ROs & 1 dosimetrist | MC |
| Lustberg, T., et al. (2018) [12] | R | LL, RL, H, O, SC, M | Mirada DLC  Expert | 470  (T= 450, V= NR, ET= 20) | 1 RO | MC using guidelines & atlas-based contours |
| Nemoto, T., et al. (2020) [10] | R | CL | 2D and 3D U-Net | 232 NSCLC  (T= 160, V= 40, ET= 32) | 2 ROs | MC using guidelines & atlas-based contours |
| Vaassen, F., et al. (2022) [24] | R | LL, RL, H, O, SC, M | Mirada DLC  Expert | 350  (T= 200, V= NR, ET= NR) | Multiple | MC |
| Vaassen, F., et al. (2021) [25] | R | LL, RL, H, O, SC, M | Mirada DLC  Expert | 20 stage I-III NSCLC  (T= NR, V= NR, ET= NR) | 1 RT | MC |
| Vu, C. C., et al. (2020) [26] | R | LL, RL, H, O, SC | 2D U-Net | 168  (T= 110, V= 29, ET= 29) | Multiple | MC |
| Zhang, F., et al. (2022) [31] | R | LL, RL, H, O, SC, Tr | DenseNet | 36 NSCLC  (T= 27, V= 9, ET= 6) | Multiple | MC |
| Zhang, T., et al. (2020) [22] | R | LL, RL, H, O, SC | ResNet-101 | 269 NSCLC  (T= 200, V= 50, ET= 19) | Multiple ROs | MC using guidelines & atlas-based contours |
| Zhu, J., et al. (2019) [7] | R | LL, RL, H, O, SC | DeepLabv3+ | 60  (T= 48, V= NR, ET= 12) | 3 ROs | MC using guidelines |
| Zhu, J., et al. (2019) [15] | R | CL, H, O, SC | 3D U-Net | 66  (T= 30, V= NR, ET= 36) | 3 ROs | MC consensus using STAPLE algorithm & atlas-based contours |
| Maduro Bustos, L. A., et al. (2023) [33] | R | LL, RL, H, O, SC | A commercially available deep image-to-image algorithm | 10  (T= NR, V= NR, ET= NR) | 1 Medical physicist & 2 dosimetrists | MC |
| Pan, S., et al. (2023) [34] | R | LL, RL, H, SC | MLP-Mixer Vnet | 90  (T= 54, V= NR, ET= 36) | 2 Physicians | MC |
| Saha, M., et al. (2023) [35] | R | LL, RL, H, O | U-Net and nnU-Net | 118  (T= 90, V= NR, ET= 28) | 1 RT | MC |
| Zhang, F., et al. (2023) [38] | R | LL, RL, H, O, SC, Tr | Two step method using DenseNet and a residual attention network | 59  (T= 39, V= 8, ET= 12) | 1 RO | MC |
| Harten, L., et al. (2019) [41] | R | H, O, Tr | 2D and 3D CNNs | 60 NSCLC  (T= 35, V=5, ET=20) | NR | MC using guidelines |
| Vesal, S., et al. (2019) [46] | R | H, O, Tr | 2D U-Net+DR | 60 NSCLC  (T= 32, V=8, ET=20) | 1 Radiologist | MC |
| Han, M., et al. (2019) [40] | R | H, O, Tr | VB-Net | 60 NSCLC  (T= 32, V=8, ET=20) | NR | MC |
| Gibbons, E., et al.  (2023) [11] | R | LL, RL, H, O | Mirada DLC  Expert | 602  (T= 572, V= NR, ET= 30) | 1 RO & 5 RTs | MC using guidelines |
| Chen, W., et al.  (2021) [39] | R | LL, RL, H, O, SC | AiContour (based on UNet) | 15  (T= NR, V=NR, ET= 15) | 1 RO | MC |
| Ribeiro, M., et al.  (2023) [44] | R | LL, RL, H, O | 3D U-Nets | 122  (T= 80, V=19, ET=23) | 1 RO | MC |
| Yang, J., et al.  (2018) [47] | R | LL, RL, H, O, SC | 2D, 2.5D and 3D UNets, 2D ResNet & 2D Multiclass network | 60  (T=36, V= NR, ET= 24) | Multiple | MC using guidelines |
| Mehta, A., et al.  (2023) [48] | R | LL. RL. H, SC | U-Net | 23  (T=18, V= 2, ET= 3) | NR | MC |

R: retrospective, P: prospective, CTV: clinical target volume, GTV: gross target volume, 2D: two-dimensional, 3D: three-dimensional, RO: radiation oncologist, NSCLC: non-small cell lung cancer, SCLC: small cell lung cancer, ES: early stage, LA: locally advanced, MC: manual contours, CNN: convolutional neural network, T: training, V: validation, ET: end-testing, SBRT: stereotactic body radiation therapy, RT: radiation therapist, STAPLE: simultaneous truth and performance level estimation NA: not applicable, NR: not reported, DLC: deep learning contouring, MLP: multi-layer perceptron, LL: left lung, RL: right lung, H: heart, O: oesophagus, SC: spinal cord, Tr: trachea, M: mediastinum, CL: combined lung

**Table 2: Studies evaluating the accuracy, variability and contouring time.**

| **Study Author (Year), [Reference]** | **Target volume or organ at risk** | **Metrics** | **DSC** | **Hausdorff Distance** | **Variability** | **Contouring time** |
| --- | --- | --- | --- | --- | --- | --- |
| Bi, N., et al. (2019) [17] | CTV | Accuracy: mean DSC  Variability: CV and SDD  Contouring time: minutes | DSC: 0.75 ± 0.06 | N/R | DL:  CV= (mean ± SD: 0.129 ± 0.040)  SDD= (mean ± SD: 0.47 ± 0.22mm)  Manual:  CV= (mean ± SD: 0.183 ± 0.043)  SDD= (mean ± SD: 0.72 ± 0.41mm) | DLAC: 9.59 minutes [IQR 7.64-11.91]  Manual: 14.81 minutes [IQR 11.88-18.64] |
| Hosny, A., et al. (2022) [21] | GTV | Variability: DSC and surface dice  Contouring time: minutes | N/R | N/R | DL:  DSC= 0·91 [IQR 0·83–0·92]  Surface dice= 0·86 [IQR 0·71–0·91]  Manual:  DSC= 0·83 [IQR 0·77–0·88]  Surface dice= 0·72 [IQR 0·61–0·81] | DLAC: 5.4 minutes  Manual: 15·5 minutes |
| Lei, Y., et al. (2022) [23] | GTV | Accuracy: DSC and HD95 | DSC: 0.84-0.88 ± 0.15 | HD95: 3.06-4.6 mm | N/R | N/R |
| Wang, C., et al. (2019) [27] | GTV | Accuracy: DSC  Contouring time: seconds and minutes | DSC: 0.82 ± 0.10 | N/R | N/R | Inference time: 30.0 seconds  DLAC: 10-30 minutes |
| Wang, S., et al. (2022) [2] | GTV | Accuracy: mean DSC and HD | Mean DSC: 0.83 ± 0.07 | HD: 5.9 ± 2.5 mm | N/R | N/R |
| Xie, Y., et al. (2021) [29] | CTV | Accuracy: DSC and HD  Contouring time: seconds and minutes | DSC: 0.86 ± 0.03 | HD: 5.5-8.6 mm | N/R | Inference time: 3.8-4.0 seconds  Manual: 5-20 minutes |
| Yu X., et al. (2022)[30] | GTV | Accuracy: DSC and HD95  Contouring time: seconds | DSC: 0.74 | HD95: 21.39 mm | N/R | Inference time: 3.0 seconds |
| Zhong Z., et al. (2019)[14] | GTV | Accuracy: DSC | DSC: 0.86 ± 0.04 | N/R | N/R | N/R |
| Yu, X., et al. (2023) [36] | GTV | Accuracy: DSC  Contouring time: seconds | DSC: 0.84 ± 0.06 | N/R | N/R | Inference time: 1.9 seconds |
| Zhang, F., et al. (2024) [37] | GTV | Accuracy: DSC and HD95 | DSC: 0.80 ± 0.13 | HD95: 9.43 mm | N/R | N/R |
| Primakov, S. P., et al.  (2022) [43] | GTV | Accuracy: DSC and HD95  Variability: DSC  Contouring time: seconds | DSC: 0.85 ± 0.15 | HD95: 5 mm | DL:  VD= 0.82 [IQR= 0.14]  Manual:  VD= 0.84 [IQR= 0.12] | Inference time: 2.9 seconds  Manual: 172.19 seconds |
| Shen, J., et al. (2022) [45] | CTV | Accuracy: DSC and HD95  Contouring time: minutes | DSC: 0.81 | HD95: 1.61-4.68 mm | N/R | Inference time: 5 minutes  DLAC: 25 minutes  Manual: 80 minutes |
| Kulkarni, C., et al.  (2024) [42] | GTV | Accuracy: DSC | DSC: 0.77 (0.0-0.93) | N/R | N/R | N/R |
| Wong J., et al. (2021) [28] | GTV  LL, RL, H, O, SC, Tr | Accuracy: mean DSC and HD95 | Mean DSC:  GTV= 0.71 (0.19–0.90)  LL= 0.98 (0.92–0.99)  RL= 0.98 (0.96–0.99)  H= 0.95 (0.87–0.98)  O= 0.81 (0.64–0.96)  SC= 0.9 (0.74–0.98)  Tr= 0.91 (0.79–0.98) | HD95:  GTV= 5.23 mm (2.04–15.17)  LL= 2.93 mm (1.97–6.73)  RL= 3.04 mm (1.26–5.40)  H= 5.09 mm (2.54–8.55)  O= 3.32 mm (2.05-6.94)  SC= 1.62 mm (0.56-2.69)  Tr= 2.27 mm (1.09-3.80) | N/R | N/R |
| Cao, Z., et al. (2021) [18] | LL, RL, H, O, SC, Tr | Accuracy: DSC  Contouring time: seconds | DSC:  LL= 0.97  RL= 0.97  H= 0.95  O= 0.85  SC= 0.91  Tr= 0.81 | N/R | N/R | Inference time: 4.0-58.0 seconds |
| Dong, X., et al. (2019) [19] | LL, RL, H, O, SC | Accuracy: DSC and HD95  Contouring time: seconds | DSC:  LL= 0.97 ± 0.01  RL= 0.97 ± 0.01  H= 0.87 ± 0.05  O= 0.75 ± 0.08  SC= 0.90 ± 0.04 | HD95:  LL= 2.07 mm ± 1.93  RL= 2.50 mm ± 3.34  H= 4.58 mm ± 3.67  O= 4.52 mm ± 3.81  SC= 1.19 mm ± 0.46 | N/R | Inference time: 6.0 seconds |
| Feng, X., et al. (2019) [20] | LL, RL, H, O, SC | Accuracy: DSC and HD95  Variability: normalisation scores  Contouring time: minutes | DSC:  LL= 0.98 ± 0.01  RL= 0.97 ± 0.02  H= 0.93 ± 0.02  O= 0.73 ± 0.1  SC= 0.89 ± 0.04 | HD95:  LL= 2.10 mm ± 2.94  RL= 3.96 mm ± 2.85  H= 6.57 mm ± 1.50  O= 8.71 mm ± 10.59  SC= 1.89 mm ± 0.63 | LL= 79.5 ± 8.96  RL= 71.39 ± 19.78  H= 46.87 ± 11.06  O= 27.6 ± 20.89  SC= 61.17 ± 14.14  Final average normalisation score= 57.31 | Inference time: 1 minute  DLAC: 7.5 ± 2.4 minutes  Manual: 30-40 minutes |
| Francis, S., et al. (2022) [3] | LL, RL, H, O, SC | Accuracy: DSC and HD95  Contouring time: seconds | DSC:  LL= 0.98 ± 0.01  RL= 0.97 ± 0.02  H= 0.94 ± 0.01  O= 0.74 ± 0.08  SC= 0.90 ± 0.02 | HD95:  LL= 1.47 mm ± 0.51  RL= 2.70 mm ± 2.19  H= 3.63 mm ± 0.94  O= 1.85 mm ± 0.43  SC= 6.65 mm ± 7.47 | N/R | Inference time: 8.63 seconds |
| Johnston, N., et al. (2022) [22] | CL, H, O, SC, Tr | Accuracy: DSC and HD95 | DSC:  CL= 0.98 ± 0.01  H= 0.91 ± 0.06  O= 0.72 ± 0.15  SC= 0.80 ± 0.06  Tr= 0.84 ± 0.06 | HD95:  CL= 6.9 mm  H= 17.6mm  O= 11.6mm  SC= 29.6mm  Tr= 11.8mm | N/R | N/R |
| Lustberg, T., et al. (2018) [12] | LL, RL, H, O, SC, M | Accuracy: DSC and HD  Contouring time: minutes | DSC:  LL= 0.98  RL= 0.98  H= 0.93  O= 0.76  SC= 0.83  M= 0.95 | HD:  LL= 3 mm  RL= 4 mm  H= 14 mm  O= 6 mm  SC= 4 mm  M= 7 mm | N/R | DLAC: 10 minutes  Atlas-based AC: 12.2 minutes  Manual: 20 minutes |
| Nemoto, T., et al. (2020) [10] | CL | Accuracy: mean DSC | DSC:  CL= 0.990 ± 0.004 | N/R | N/R | N/R |
| Vaassen, F., et al. (2022) [24] | LL, RL, H, O, SC, M | Accuracy: DSC and MSHD | DSC:  LL= 1.0 (0.99–1.0)  RL= 1.0 (0.99–1.0)  H= 0.89 (0.77–0.94)  O= 0.73 (0.32–0.89)  SC= 1.00 (0.93–1.00)  M= 0.93 (0.85–0.96) | MSHD:  LL= 0.9 mm (0.0-3.4)  RL= 1.1 mm (0.0-5.0)  H= 11.3 mm (5.8-21.8)  O= 3.2 mm (1.5-5.7)  SC= 0 mm (0.0-1.3)  M= 9.9 mm (6.4-16.2) | N/R | N/R |
| Vaassen, F., et al. (2021) [25] | LL, RL, H, O, SC, M | Accuracy: DSC and MSHD | Exact figures not provided | Exact figures not provided | N/R | N/R |
| Vu, C. C., et al. (2020) [26] | LL, RL, H, O, SC | Accuracy: DSC and HD95  Contouring time:  seconds | DSC:  DL  LL= 0.97  RL= 0.97  H= 0.90  O= 0.64  SC= 0.75  Atlas-based  LL= 0.91  RL= 0.93  H= 0.83  O= 0.39  SC= 0.63 | HD95:  DL  LL= 4 mm  RL= 5.1 mm  H= 9.8 mm  O= 9.2 mm  SC= 9.5 mm  Atlas-based  LL= 8 mm  RL= 8.1 mm  H= 15.8 mm  O= 20 mm  SC= 25.3 mm | N/R | Inference time: 15.0 seconds |
| Zhang, F., et al. (2022) [31] | LL, RL, H, O, SC, Tr | Accuracy: DSC and HD95  Contouring time:  seconds and minutes | DSC:  LL= 0.95 ± 0.02  RL= 0.96 ± 0.01  H= 0.86 ± 0.09  O= 0.67 ± 0.12  SC= 0.89 ± 0.01  Tr= 0.91 ± 0.03 | HD95:  LL= 6.47 mm ± 3.27  RL= 6.09 mm ± 1.56  H= 9.75 mm ± 2.34  O= 6.14 mm ± 3.07  SC= 2.05 mm ± 0.38  Tr= 2.44 mm ± 1.17 | N/R | Inference time: 0.17 seconds  DLAC: 9 minutes  Manual: 15.2 minutes |
| Zhang, T., et al. (2020) [22] | LL, RL, H, O, SC | Accuracy: DSC  Contouring time: minutes | DSC:  DL  LL= 0.95 ± 0.01  RL= 0.94 ± 0.02  H= 0.89 ± 0.05  O= 0.73 ± 0.07  SC= 0.82 ± 0.05  Atlas-based  LL= 0.93 ± 0.04  RL= 0.94 ± 0.02  H= 0.86 ± 0.08  O= not available  SC= 0.87 ± 0.03 | N/R | N/R | Inference times:  DL=1.6 minutes  Atlas-based= 2.4 minutes  Manual: 25.4 minutes |
| Zhu, J., et al. (2019) [7] | LL, RL, H, O, SC | Accuracy: DSC  Variability: DSC  Contouring time: minutes | DSC:  LL= 0.95-0.97  RL= 0.96-0.97  H= 0.91-0.94  O= 0.75-0.81  SC= 0.87-0.89 | N/R | LL: 0.95-0.97  RL: 0.96-0.97  H: 0.94-0.95  O: 0.79-0.84  SC:0.87-0.89 | DLAC: 15–20 minutes  Manual: 40–50 minutes |
| Zhu, J., et al. (2019) [15] | CL, H, O, SC | Accuracy: DSC and HD95  Variability: DSC  Contouring time: minutes | DSC:  DL  CL= 0.95 0.01  H= 0.91 ± 0.03  O= 0.64 ± 0.09  SC= 0.76 ± 0.04  Atlas-based  CL= 0.95 0.01  H= 0.90 ± 0.04  O= 0.54 ± 0.08  SC= 0.71 ± 0.06 | HD95:  DL  CL= 7.96 mm ± 2.57  H= 7.98 mm ± 4.56  O= 9.25 mm ± 5.60  SC= 8.74 mm ± 6.60  Atlas-based  CL= 8.07 mm ± 2.39  H= 9.53 mm ± 4.99  O= 9.45 mm ± 4.64  SC= 11.97 mm ± 6.88 | CL: Experts= 0.95 0.01  CL: CNN= 0.95 0.01  H: Experts= 0.94 ± 0.03  H: CNN= 0.92 ± 0.02  O: Experts= 0.82 ± 0.06  O: CNN= 0.71 ± 0.05  SC: Experts= 0.79 ± 0.07  SC: CNN= 0.80 ± 0.03 | Inference time:  DL= 0.2 minutes  Atlas-based= 3 minutes |
| Maduro Bustos, L. A., et al. (2023) [33] | LL, RL, H, O, SC | Accuracy: mean DSC and HD  Contouring time: hours | DSC:  LL= 0.95  RL= 0.96  H= 0.95  O= 0.75  SC= 0.83 | HD:  LL= 12.5 mm  RL= 12 mm  H= 10.1 mm  O= 21 mm  SC= 4.2 mm | N/R | Total time saved: 5.4 hours (84%) |
| Pan, S., et al. (2023) [34] | LL, RL, H, SC | Accuracy: DSC and HD  Contouring time:  seconds | DSC:  LL= 0.97 ± 0.02  RL= 0.98 ± 0.01  H= 0.90 ± 0.11  SC= 0.89 ± 0.07 | HD:  LL= 2.64 mm  RL= 7.03 mm  H= 8.21 mm  SC= 8.77 mm | N/R | Inference time: 4.03 seconds |
| Saha, M., et al. (2023) [35] | LL, RL, H, O | Accuracy: mean DSC and HD  Contouring time:  minutes | DSC:  LL= 0.96 ± 0.02  RL= 0.97 ± 0.01  H= 0.94 ± 0.02  O= 0.61 ± 0.10 | HD:  LL= 0.8 mm  RL= 0.7 mm  H= 2 mm  O= 1 mm | N/R | Inference time: 9 minutes |
| Zhang, F., et al. (2023) [38] | LL, RL, H, O, SC, Tr | Accuracy: DSC and HD95 | DSC:  LL= 0.94 ± 0.02  RL= 0.92 ± 0.03  H= 0.89 ± 0.03  O= 0.73 ± 0.06  SC= 0.87 ± 0.01  Tr= 0.81 ± 0.03 | HD95:  LL= 10.77 mm ± 3.25  RL= 11.92 mm ± 4.56  H= 8.89 mm ± 3.1  O= 4.32 mm ± 1.02  SC= 7.09 mm ± 0.38  Tr= 3.78 mm ± 0.87 | N/R | N/R |
| Harten, L., et al. (2019) [41] | H, O, Tr | Accuracy: DSC and HD | DSC:  H= 0.84 ± 0.05  O= 0.94 ± 0.02  Tr= 0.91± 0.02 | HD:  H= 3.4 mm ± 2.3  O= 2.0 mm ± 1.1  Tr= 2.1 mm ± 1.0 | N/R | N/R |
| Vesal, S., et al. (2019) [46] | H, O, Tr | Accuracy: DSC and HD | DSC:  H= 0.94  O= 0.86  Tr= 0.93 | HD:  H= 0.33 mm  O= 0.23 mm  Tr= 0.19 mm | N/R | N/R |
| Han, M., et al. (2019) [40] | H, O, Tr | Accuracy: DSC and HD  Contouring time: seconds | DSC:  H= 0.95  O= 0.87  Tr= 0.93 | HD:  H= 1.3 mm  O= 2.6 mm  Tr= 1.5 mm | N/R | Inference time: 2.01 seconds |
| Gibbons, E., et al.  (2023) [11] | LL, RL, H, O | Accuracy: DSC and HD  Contouring time: minutes | DSC:  DL  LL= 0.98  RL= 0.98  H= 0.96  O= 0.74  Atlas-based  LL= 0.98  RL= 0.98  H= 0.96  O= 0.48 | HD:  DL  LL= 18.9 mm  RL= 17 mm  H= 17.6 mm  O= 12.4 mm  Atlas-based  LL= 22.3 mm  RL= 22.5 mm  H= 21 mm  O= 18.8 mm | N/R | DLAC= 6.7 minutes  Atlas-based= 8.6 minutes  Manual: 10.3 minutes |
| Chen, W., et al.  (2021) [39] | LL, RL, H, O, SC | Accuracy: DSC | DSC:  DL  LL= 0.98  RL= 0.98  H= 0.93  O= 0.75  SC= 0.90  Atlas-based  LL= 0.96  RL= 0.96  H= 0.86  O= 0.40  SC= 0.58 | N/R | N/R | N/R |
| Ribeiro, M., et al.  (2023) [44] | LL, RL, H, O | Accuracy: DSC and HD95 | DSC:  LL= 0.96  RL= 0.96  H= 0.94  O= 0.78 | HD95:  LL= 1.4 mm  RL= 1.6 mm  H= 1.8 mm  O= 1.2 mm | N/R | N/R |
| Yang, J., et al.  (2018) [47] | LL, RL, H, O, SC | Accuracy: DSC and HD95  Variability: normalisation scores  Contouring time: seconds, minutes and hours | DSC:  DL  LL= 0.95-0.98  RL= 0.95-0.97  H= 0.85-0.93  O= 0.55-0.72  Sc= 0.83-0.89  Atlas-based  LL= 0.96-0.97  RL= 0.96-0.97  H= 0.90  O= 0.58-0.64  SC= 0.87-0.88 | HD95:  DL  LL= 2.9-7.8 mm  RL= 4.1-14.5 mm  H= 5.8-13.8 mm  O= 7.3-37 mm  SC= 1.9-8.1 mm  Atlas-based  LL= 3-4.5 mm  RL= 4.6-5.6 mm  H= 9.2-9.9 mm  O= 6.8-8.6 mm  SC= 2-2.1 mm | Final average normalisation scores:  DL  35.19-56.32  Atlas-based  36.13-48.86 | Inference times:  DL  10 seconds - 6 minutes  Atlas-based  5 minutes - 8 hours |
| Mehta, A., et al.  (2023) [48] | LL, RL, H, SC | Accuracy: DSC and HD | DSC:  LL= 0.94  RL= 0.92  H= 0.88  SC= 0.74 | HD:  LL= 3.6 mm  RL= 4.1 mm  H= 4.2 mm  SC= 2.85 mm | NR | NR |

CTV: clinical target volume, GTV: gross target volume, DSC: dice similarity coefficient, HD: Hausdorff distance, HD95: 95th percentile Hausdorff distance, CV: coefficient of variation, SDD: standard distance deviation, SD: standard deviation, MM: milimetres, DL: deep learning, DLAC: deep learning adjusted contour, AC: adjusted contour IQR: interquartile range, N/R: not reported, CNN: convolutional neural network, LL: left lung, RL: right lung, H: heart, O: oesophagus, SC: spinal cord, Tr: trachea, M: mediastinum, CL: combined lung
